# Reading Rate and Comprehension for Text Presented on Tablet and Paper: Evidence from Arabic

**DOI:** 10.3389/fpsyg.2017.00257

**Published:** 2017-02-21

**Authors:** Ehab W. Hermena, Mercedes Sheen, Maryam AlJassmi, Khulood AlFalasi, Maha AlMatroushi, Timothy R. Jordan

**Affiliations:** Cognition and Neuroscience Research Laboratory, Department of Psychology, Zayed UniversityDubai, UAE

**Keywords:** reading Arabic, reading from tablets, luminance levels in reading, reading media, reading technology, reading comprehension

## Abstract

The effectiveness of tablet computers to supplement or replace paper-based text in everyday life has yet to be fully revealed. Previous investigations comparing reading performance using tablets and paper have, however, reported inconsistent results. Furthermore, the interpretability of some previous findings is limited by lack of experimental control over variables like text display conditions. In the current study, we investigated reading performance for text presented on tablet and paper. Crucially, the levels of luminance and contrast were matched precisely across tablet and paper. The study used Arabic text which differs substantially from the languages used previously to investigate effects of tablet and paper on reading, thus offering a distinctive test of the influence of these two media on reading performance. The results suggest that when text display conditions are well-matched, there is no reliable difference in reading performance between the two media. Also, neither the order of medium (reading from tablet or paper first), nor familiarity with using a tablet significantly influence reading performance. These results call into question previous suggestions that reading from tablets is linked to poorer reading performance, and demonstrate the benefits of controlling text display conditions. These findings are of interest to reading scientists and educators.

## Introduction

Reading from tablets and tablet-like devices (e.g., iPad, Galaxy, Kindle, for which we shall use the generic term tablets), whether for personal enjoyment, conducting business, or educational purposes, is becoming progressively more common, especially given the availability and increased functionality of these devices (e.g., Connell et al., 2012). Using such non-paper based media for reading has particularly attracted the attention of educators, given that such non-traditional methods have been suggested to increase students’ motivation to read and engage with the texts being read (Horney and Anderson-Inman, 1999; Korat and Shamir, 2007). Research activity into the efficacy of using tablets, relative to traditional paper books, has thus far shed some light on the similarities and differences between the two modes of reading (e.g., Grimshaw et al., 2007). However, as we shall discuss further, the results from previous investigations can be described as mixed, and the generalizability of the results remains questionable due to lack of control over crucial experimental variables. In the current investigation, we aim to address these shortcomings by providing clear answers to specific questions regarding reading from tablets compared to paper, and by controlling the conditions under which reading from both media was conducted. Additionally, the current investigation is, to our knowledge, the first to compare these reading media for reading Arabic text. Our findings will thus expand on existing findings in this area where, with few exceptions, the focus of research remains on English and other European languages.

Using electronic media was found to improve reading comprehension (assessed by multiple choice questions), relative to paper-based reading (de Jong and Bus, 2002; Grimshaw et al., 2007; Korat, 2009). Such findings were further seen as evidence that tablets improve readers’ motivation and engagement (e.g., Grimshaw et al., 2007), particularly in children who have struggled to (or experienced failure in) learning to read (de Jong and Bus, 2004; Gleason, 2005; Korat, 2009; Shamir, 2009; Black, 2010). Furthermore, and in addition to clearly displaying text, tablets typically include other learning resources such as the ability to access dictionary and thesaurus, being equipped with features such as reading words or text out loud, and the ability to annotate text and highlight it. As a result, the adoption of these technologies was further seen by researchers and teachers as a means to improve the pedagogical input of teachers in class, making it more efficient and tailored to suit the learners’ need for support, as well as independence (e.g., Kinzer, 2003; Prensky, 2006; Bennett et al., 2008; Larson, 2008, 2009).

However, the potential benefits of tablets for reading have come under doubt from studies that suggest that reading time is longer when reading from tablets compared to when reading the same text from paper (e.g., Grimshaw et al., 2007; Nielsen, 2010; Schugar et al., 2011; Connell et al., 2012). For instance, Nielsen (2010) reported that readers were on average about 6 to 11% slower when reading from tablets compared to when they read the same text from paper (for similar results, see Mayes et al., 2001; Wästlund et al., 2005; Ackerman and Goldsmith, 2011; Kim and Kim, 2013; Mangen et al., 2013; Mangen and Kuiken, 2014). Other investigations of reading in English reported no differences between the reading media, tablet or paper, in reading comprehension, but replicated the finding that reading from tablet is significantly slower than reading from paper (Connell et al., 2012). Some researchers have also suggested that reading from electronic displays may be slower because these displays are brighter (of higher luminance) than paper, and that this increased brightness results in greater eye fatigue (e.g., Blanco and Leirøs, 2000; see also Dundar and Akcayir, 2012). Indeed, other evidence suggests that, when processing visual stimuli, luminance levels influence reaction times and the allocation of spatial attention (e.g., Johannes et al., 1995; Kammer et al., 1999). It is also likely that the difference in reading time between tablet and paper in some studies was due to insufficient control of fundamental variables, whereby font sizes and types, number of pages of text, and even the amount of text per page differed between the two reading media (see e.g., Mangen and Kuiken, 2014). Another point of concern regarding previous investigations relates to the use of self-report by participants concerning how much reading they did on tablet and paper, and researchers actually losing track of the amounts of reading done using these media when reporting or interpreting reading rate and comprehension results. For instance, Schugar et al. (2011, p. 183) reported that: “Additionally as the study progressed, *it was not always obvious who was reading what on what*, and while those students who used the device did some of their reading on the device, some students owned both digital and print based versions of the text.” (Emphasis added).

The increased reading times from tablets compared to paper has also been linked to readers’ lack of familiarity with how to operate the tablet, or with how to access its features (e.g., Wright et al., 2013). Chen et al. (2014) investigated if familiarity with tablet operation influenced reading performance when comparing reading in Chinese from paper, computer screen, and tablets. They used a questionnaire to evaluate readers’ familiarity with tablets (Tablet Familiarity Questionnaire, TFQ: Zheng et al., 2015) which explores areas such as overall perceived ease or difficulty of tablet use, ease of performing tasks such as editing text, reading, browsing the web, troubleshooting (fixing problems), watching or listening to media, contacting friends using the tablet, installing or removing applications, etc. Chen et al. (2014) found that participants’ level of familiarity with tablet operation influenced their deep comprehension (ability to summarize text after reading) such that participants who were more familiar with the tablet operation were significantly more successful in reading comprehension. The majority of reported investigations in this area have failed to take into account user tablet familiarity when presenting and interpreting their results.

As mentioned above, the evidence available thus far is mixed with regards to the influence of the reading medium, tablet or paper, on reading performance. Some investigations (e.g., those reported above) found that reading from tablets is slower than reading from paper. By contrast, other studies reported that there were no significant differences between measures of reading speed and text comprehension when participants were required to read the same text from paper or from a tablet in English (e.g., Noyes and Garland, 2003, 2008). In Italian, a study by Zambarbieri and Carniglia (2012) found that there was no difference between eye movement behaviors (e.g., fixation duration, and the amount of forward and backward eye movements, or saccades, in the text) while reading from a tablet or paper (although fixation durations were longer when participants read from the fourth medium investigated, namely a computer screen). Dundar and Akcayir (2012) reported similar results in reading Turkish text from tablet and paper, with no significant differences in reading rate or comprehension between the two media. Unfortunately, without exerting proper control over the parameters of the displays used for each medium, it is impossible to determine whether text is or is not read equally efficiently on tablets and paper.

The current investigation addressed some major shortcomings in previous studies of reading from tablet and paper. To begin with, we adopted a methodical approach in matching the visual properties of the text presentation by matching the luminance and contrast levels of tablet and paper displays. This allowed us to avoid the potentially confounding influence of varying these crucial components of each display. Additionally, we investigated whether the order of medium (reading first from a tablet vs. from paper) had any influence on readers’ performance. It is plausible that readers who begin their reading session by reading from a tablet are more motivated or engaged in the task given the novelty of the tablet medium (e.g., Grimshaw et al., 2007), relative to those who begin the reading session from paper. If this were the case, we may be able to observe differences in reading performance between participants who begin reading with tablet and then switch to paper and the group that read from both media in the opposite order. Furthermore, we also systematically investigated the participants’ level of familiarity with tablets, given the documented influence of this variable on reading performance.

Finally, we studied reading Arabic in both these media given that it has thus far not been investigated. Arabic has the second-most used alphabet in human societies, after the Latinate alphabet. But, unlike languages using the Latinate alphabet, Arabic is formed in cursive script in which clear spaces often do not exist between letters in words, even when formally printed, and this may present problems for word recognition (see Jordan et al., 2010; Paterson et al., 2015). Indeed, the physical shapes of Arabic letters also vary considerably depending on their position in words, and these variations increase the total number of forms of Arabic letters to over 100. Thus, Arabic differs substantially from the languages that have been used previously to study the effects of reading text from tablet and paper and so offers a distinctive test of the relative influence of these two media on reading. Increasingly, using modern technological tools such as tablets is becoming common in the Arabic classroom (e.g., Al Bataineh and Anderson, 2015; Alresheed et al., 2015), and the current investigation should be regarded as a step toward building an evidence base for the current practices and possible necessary revisions of such practices.

Thus, to summarize, we addressed the following questions: (a) Is reading from tablet slower than reading from paper, even if the visual properties of the display in both conditions are matched? (b) Does the order of presenting the reading materials on each medium (tablet first or paper first) influence reading performance? (c) To what extent does users’ familiarity with the tablet predict, or modulate, their reading rate and comprehension? (d) And finally, given the absence of evidence about reading Arabic text from tablet or paper, our investigation would reveal, for the first time, if reading Arabic text differs in terms of rate and comprehension because of the reading medium. As discussed above, findings from previous investigations are mixed, possibly due to lacking the adequate controls (e.g., on text display luminance), and possibly due to failing to take into account (in statistical models) the influence of relevant factors such as participant differences in the level of familiarity with tablets. We predict that the findings from this investigation will be equally informative to reading scientists and educators.

## Materials and Methods

### Participants

Twenty-four undergraduate students from Zayed University contributed to the experiment. Participants were aged between 18 and 31 and were all native Arabic speakers. All participants had normal or corrected-to-normal vision as determined by the Snellen eye chart.

### Stimuli

The reading materials were two Arabic passages, each containing 604 words, selected from the novel Masafat by Atallah (2009). These passages were edited and adjusted to be easier for reading comprehension. An additional practice passage from the same book (200 words) was shown before the experimental passages. All passages were presented in 11-point Helvetica font, with a line spacing of 1.2 pt. After reading each passage, six multiple choice questions were presented orally to the participants. The questions referred to different aspects and details of the preceding text and were designed to ensure that participants read and comprehended each presented text. All texts and questions were presented to all participants in Modern Standard Arabic, which is spoken, understood, and read fluently by all the participants, being native Arabic readers. All presented texts contained no vowelized (diacritized) words.

A TFQ (Zheng et al., 2015) was presented to participants in order to gauge their levels of familiarity and confidence using a tablet. The questionnaire contained 30 items and the participants responded to each item by indicating on a scale from 1 to 5 how they rate their knowledge and ability to use a tablet. A response of 5 indicated high a level of tablet familiarity in some questions, whereas in other questions a response of 5 indicated a low level of tablet familiarity. As explained above, the questionnaire explored areas such as overall perceived ease of tablet use, and ease with which tasks such as editing text, reading, browsing the web, troubleshooting, watching or listening to media, etc., are performed.

### Apparatus and Text Display Conditions

Viewing the text was binocular. Testing took place in a well-lit room (natural and electric light, but no direct sunlight) and light conditions were stable for all participants and all testing sessions.

For the tablet condition, each passage was presented on an Apple iPad 3 (9.50 × 7.31 inch). For the paper condition, each passage was presented on a sheet of paper of the same size as the iPad display. In both display media, the text was presented in black on a white background, and a complete passage of text filled an area approximately 11.4° (horizontal) × 15.6° (vertical). The paper margins matched the iPad frame size (1.4 inch).

The text luminance on paper was measured in the location where testing was conducted. A Velleman DVM1300 Digital Light Meter was used to measure the luminance. The automatic luminance-setting feature on the iPad was turned off and the luminance of the iPad display was matched to that of the paper using the same instrument. This matching process was conducted before testing. For both the iPad and the paper, text luminance was 1.0 cd/m^2^ and background luminance was 18.5 cd/m^2^. These values gave a Michelson contrast ratio of 0.90 which is well-suited to reading.

All passages were viewed from a distance of 33 cm and a chinrest was used to ensure a constant viewing distance. The iPad and the paper were each mounted in turn on a table-top stand and were angled identically toward each participant for comfortable reading.

### Design

Reading medium was a within participant independent variable with two levels: iPad and paper. The order in which the reading passages were presented (passage A and passage B), as well as the reading medium (iPad or paper) from which the readers read the first passage, were counterbalanced. Accordingly, half the participants started with passage A (half with iPad first and half with paper first) and half started with passage B (half with iPad first and half with paper first). Passage reading speed (in seconds), and reading comprehension performance were the dependent variables. Another predictive variable that we also include in our statistical models is readers’ tablet familiarity score, based on their reposes in the TFQ (Zheng et al., 2015). As outlined above, readers’ familiarity with tablets may influence their reading performance, and we wanted to quantify this effect by including it as a continuous variable in our models.

### Procedure

This research was approved by the Research Ethics Committee at Zayed University. At the beginning of each testing session, participants were informed that they would be required to read passages of text and that their reading speed and comprehension would be measured. Reading time was measured from when the text was revealed and stopped when the participants indicated that they had reached the end of each passage by tapping the table. Each participant began by reading the practice passage, and the two experimental passages were then presented, one on the iPad and one on the paper. After reading each passage, participants were asked six passage-related comprehension questions by the experimenter, to which they provided simple verbal responses. Finally, participants completed the TFQ. Each experimental session lasted about 20 min.

## Results

We used the lme4 package (version 1.1-12, Bates et al., 2011) within the R environment for statistical computing (R Core Team, 2013) to run linear mixed models (LMMs). We report *t* statistics for the LMMs where effects approximately twice as large as their standard error (i.e., *t* ≥ 1.96) are interpreted as significant. For each of the dependent variables (reading time and comprehension score), the first fixed variable of all models were the reading medium (iPad vs. paper). We also included order of medium (iPad vs. paper) as another fixed variable to learn whether presenting readers with text on tablet first influences reading performance, as explained above. The final fixed variable in all models was the participants’ score on the TFQ. Including this continuous variable in the models allowed us to quantify its influence on readers’ performance. On average, participants had a tablet familiarity score of 85 out of possible full score of 150 (*SD* = 10, range = 70–104, with the majority of scores ≥80). This indicated that participants were fairly familiar with the operation of tablets. Participants and passages were included in the models as random variables.

We always began our analyses with full models (e.g., Barr et al., 2013) that included the main variables and their interactions, as well as maximal random variables structure (the intercepts of the random variables as well as their slopes varying as a function of the three-way interaction between the fixed variables). These models were systematically trimmed when failure to converge occurred, first by removing correlations between random effects, and, if necessary, also by removing their interactions. All findings reported here are from successfully converging models^1^. For each contrast we report beta values (b), standard error (SE), and *t* statistics for reading times and comprehension. We obtained almost identical results when reading times were log transformed and when they were not, and so we report the analyses conducted on the untransformed data to preserve transparency of interpretation. Prior to running the models, we used the contr.sdif function in the MASS package (Venables and Ripley, 2002) to pre-specify the contrasts between the levels of the fixed factors (iPad vs. paper, and order of medium).

### Reading Time

On average, reading times for tablet and paper were 255.2 and 262.5 s, respectively (see **Table 1**). The 7.3 s difference between the two media represented less than a 3% difference and was not statistically significant (*b* = -90.7, *SE* = 67.2, *t* = -1.4). The order of medium (iPad vs. paper) also had no significant influence on reading speed (*t* < 1, also see **Table 1**). Reading time was furthermore minimally influenced by tablet familiarity (*b* = -2.8, *SE* = 1.8, *t* = -1.6). There were no significant interactions between any of the variables (reading medium, order of medium, and tablet familiarity), all *t*s < 1.5.

**Table 1:** Descriptive statistics for passage reading time (s).

	Reading medium order		
	Paper first	Tablet first	Overall
Reading medium	Mean (*SD*)	Mean (*SD*)	Mean (*SD*)
Paper	257.8 (112.6)	252.6 (50.6)	255.2 (85.4)
Tablet	264.8 (104.1)	260.3 (49.2)	262.5 (79.6)

Following the method outlined by Dienes (2014), we calculated the Bayesian Factor to further ascertain that the obtained null result is not due to factors such as lack of statistical power. Accordingly, the reading time measure was collapsed across presentation order to arrive at the difference in average reading time between the paper and tablet conditions. An estimate of prior difference in reading time between tablet and paper was calculated from previous similar investigations (namely, Grimshaw et al., 2007 and Connell et al., 2012). The Bayesian Factor obtained was 0.19, indicating that accepting the null hypothesis is justified, and that the data set did not lack power or sensitivity.

### Reading Comprehension

All participants had a comprehension score of greater than 80%. Reading comprehension performance was identical for both reading from iPad and from paper (*t* < 1, also see **Table 2**). There were no significant effects of order of medium (*b* = 26.1, *SE* = 21.6, *t* = 1.2), or for tablet familiarity (*b* = 0.1, *SE* = 0.1, *t* = 1.1) on reading comprehension. Finally, there were no significant interactions between any of the variables (reading medium, order of medium, and tablet familiarity), all *t*s < 1.2.

**Table 2:** Descriptive statistics for passage comprehension scores (percentage).

	Reading medium order		
	Paper first	Tablet first	Overall
Reading medium	Mean (*SD*)	Mean (*SD*)	Mean (*SD*)
Paper	95.8 (7.7)	97.2 (6.6)	96.5 (7.1)
Tablet	97.2 (6.6)	95.8 (7.7)	96.5 (7.1)

The same procedure for calculating the Bayes Factor outlined above was followed for the reading comprehension measure. The estimate of prior difference in reading comprehension between tablet and paper was calculated from Chen et al. (2014) given the similarity of reading comprehension task and participant age to the current investigation. The Bayesian Factor obtain was 0.17, indicating that accepting the null hypothesis is justified, and that the data set did not lack power or sensitivity.

## Discussion

The purpose of this experiment was to investigate differences in reading performance (rate and comprehension) as a function of presenting text on a tablet (an iPad) or on paper. The first issue we addressed related to whether text reading performance differed if visual display conditions were matched between the two media. Previous investigations reported mixed results regarding the influence of reading medium on reading performance, with some investigations reporting slower reading from tablets while others reported no difference between paper and tablet. When exerting proper control over the display conditions for each medium, our results showed that there was a negligible and non-significant difference between reading times for tablet and paper, and no difference at all in comprehension performance between the two reading media. These results are the first to be reported in Arabic, and are broadly in line with previous findings that also reported no difference between tablet and paper reading performance (e.g., Noyes and Garland, 2003, 2008; Connell et al., 2012 when reading English; Dundar and Akcayir, 2012 when reading Turkish; Zambarbieri and Carniglia, 2012 when reading Italian). However, it is apparent that none of these previous investigations matched display conditions explicitly when comparing reading across media, and so the current findings overcome these important limitations.

The results obtained in the present study suggest that when visual presentation conditions are controlled, reading from tablets is no slower than reading from paper. This is in contrast to previous findings where increased reading times from tablets were reported, and where text display luminance and contrast was not matched between the two reading media (e.g., Grimshaw et al., 2007; Nielsen, 2010; Schugar et al., 2011; Connell et al., 2012). As explained above, eye fatigue from reading brighter tablet displays (relative to paper) cannot be ruled out as a reason for slower reading from tablets (see e.g., Mangen et al., 2013, who reported that readers of Norwegian reported more fatigue when reading from electronic displays). Contrastingly, in the current investigation, it is highly unlikely that readers experienced more eye fatigue due to higher levels of luminance when reading from tablets compared to paper, given the matched luminance levels that were used (see e.g., Blanco and Leirøs, 2000; also Dundar and Akcayir, 2012). Additionally, the fact that we found no evidence that reading from tablets is linked to slower reading or worse reading comprehension calls into question previous suggestions that reading from tablets increases reader distractibility and reduces focus (e.g., Wright et al., 2013, p. 368).

The second issue we addressed was whether the order of reading medium had any influence on reading performance. The present findings show that reading from tablet first did not result in noticeable differences in reading performance compared to reading from paper first. Consequently, if increased levels of overall engagement or motivation are produced when reading from tablets (e.g., Grimshaw et al., 2007), they do not appear to transfer to reading from paper, even in the same reading session.

We also investigated whether the extent to which readers’ familiarity with tablet operation influences their reading performance from the tablet. It will be recalled that Chen et al. (2014) reported that readers of Chinese who were more familiar with tablet operation were more likely to have better text comprehension (see also Zheng et al., 2015). Our results showed no support for this suggestion, or for the notion that level of familiarity with tablet operation influences reading times. It may be, therefore, that tablet familiarity affects tasks involving more tablet interaction (e.g., accessing study aids such as dictionaries, thesauruses, etc.) than normal textual reading and so tablet familiarity did not influence reading performance.

Finally, we used Arabic because it offers a distinctive test of the relative influence of tablet and paper on reading that has so far not been investigated. As discussed above, Arabic letter formation, and the way this differs contingent on where in the word a letter falls, gives Arabic unique visual characteristics which may make visual word recognition more challenging (see e.g., Jordan et al., 2010; Paterson et al., 2015). The findings suggest that although Arabic text is distinctive visually from the languages previously investigated (e.g., English, Norwegian, Turkish, and Chinese), when variables such as display luminance and contrast are controlled, processing the complexities inherent in Arabic does not interact with the medium on which the text is presented.

The findings we present here provide strong evidence that reading from tablets does not result in increased reading time or poorer reading comprehension, as has been suggested by previous studies. Instead, our results underscore the view that control over the visual properties of the display should enable parents, class teachers, and teaching assistants to ensure enjoyable reading sessions that are no more difficult than reading from books and ordinary print.

## Ethics Statement

This study was carried out in accordance with the recommendations of Zayed University Research Ethics Committee Guidelines with written informed consent from all subjects. All subjects gave written informed consent in accordance with the Declaration of Helsinki. The protocol was approved by the Zayed University Research Ethics Committee.

## Author Contributions

MAJ, KAF, and MAM collected data and prepared preliminary analysis. EH prepared the submitted analyses and the first draft of the manuscript. EH, TJ, and MS prepared the submitted version of the manuscript.

## Conflict of Interest Statement

The authors declare that the research was conducted in the absence of any commercial or financial relationships that could be construed as a potential conflict of interest.

## Footnotes

1 The converging model for reading time was: lmer(read_time ∼ condition ∗ order ∗ tablet_familiarity + (0 + tablet_familiarity | participants) + (1 + order ∗ condition | items), data = datafile) For the measure of reading comprehension, the converging model was: lmer(comprehension ∼ condition ^∗^ order ^∗^ tablet_familiarity + (0 + tablet_familiarity | participants) + (1 + condition | items), data = datafile)
